# Activation of Purinergic P2Y2 Receptor Protects the Kidney Against Renal Ischemia and Reperfusion Injury in Mice

**DOI:** 10.3390/ijms252312563

**Published:** 2024-11-22

**Authors:** Kyuho Jeong, Jihyun Je, Theodomir Dusabimana, Jacques Karekezi, Tatang Aldi Nugroho, Edvard Ntambara Ndahigwa, Seung Pil Yun, Hye Jung Kim, Hwajin Kim, Sang Won Park

**Affiliations:** 1 Department of Pharmacology, Institute of Medical Sciences, College of Medicine, Gyeongsang National University, Jinju 52727, Republic of Korea; khjeong@dongguk.ac.kr (K.J.); jeri1984@naver.com (J.J.); odomy2020@gmail.com (T.D.); jacqueskarekezi@gmail.com (J.K.); aldinugroho178@gmail.com (T.A.N.); ndahigwaedvard@gmail.com (E.N.N.); spyun@gnu.ac.kr (S.P.Y.); hyejungkim@gnu.ac.kr (H.J.K.); 2 Department of Biochemistry, College of Medicine, Dongguk University, Gyeongju 38066, Republic of Korea; 3 Department of Convergence Medical Science, Gyeongsang National University Graduate School, Jinju 52727, Republic of Korea

**Keywords:** acute kidney injury, ATP, purinergic P2Y2 receptor, renal ischemia and reperfusion, proximal tubular cells

## Abstract

Extracellular ATP plays an important role in renal physiology as well as the pathogenesis of acute kidney injury induced by renal ischemia and reperfusion (IR). Expression of the purinergic P2Y2 receptor has been shown on inflammatory and structural cells of the kidney, and P2Y2R is preferably activated by ATP (or UTP). Here, we investigated the molecular mechanism of P2Y2R during IR injury by using P2Y2R knockout (KO) mice and a selective P2Y2R agonist, MRS2768. After renal IR, P2Y2R KO mice showed greater increases in plasma creatinine, tubular damage and neutrophil infiltration, and significant induction of proinflammatory cytokines and apoptotic markers than wild-type (WT) mice. In contrast, treatment with MRS2768 reduced plasma creatinine levels, tubular damage and inflammation, and renal apoptosis in mice subjected to renal IR. In cultured human proximal tubular HK-2 cells, MRS2768 upregulated P2Y2R mRNA levels and decreased TNF-α/cycloheximide-induced apoptosis and inflammation. Importantly, P2Y2R activation by MRS2768 increased the phosphorylation of protein kinase C (PKC), Src, and phosphatidylinositol 3-kinase (PI3K)/Akt. In addition, the inhibition of PI3K/Akt abolished the protective effects of MRS2768 against TNF-α/cycloheximide-induced apoptosis and inflammation in HK-2 cells. In conclusion, activation of P2Y2R protects against tubular apoptosis and inflammation during renal IR via the PKC/Src/Akt pathway, suggesting P2Y2R is a promising therapeutic target for acute kidney injury.

## 1. Introduction

Acute kidney injury (AKI) is a major clinical problem associated with high rates of morbidity and mortality in hospitalized patients and increasing healthcare costs worldwide [1,2]. Renal ischemia and reperfusion (IR) injury is a major cause of AKI, and the pathophysiology of renal IR involves a complex process of tubular cell death and inflammation [3,4]. IR injury is initiated with a burst of oxidative stress and inflammatory response, leading to apoptotic or necrotic cell death, severe tubular and microvascular damage, and ultimately impaired kidney function [3]. Particularly, the proximal tubules are susceptible to IR because they are highly dependent on mitochondrial ATP for reabsorbing ~65% of filtered amino acids, solutes, and small proteins and 80% of the filtered bicarbonates in the maintenance of body electrolyte and fluid balance [5]. Thus, the ATP depletion and redox imbalance induced by IR precipitate renal injury and delay repair processes [6,7].

ATP is released to act physiologically as an extracellular second messenger or pathologically as a danger signal [8]. The extracellular ATP activates ligand-gated P2X and G-protein-coupled P2Y receptors to regulate diverse cellular functions. P2X (P2X1-7) receptors present important regulatory functions in tubular and vascular proliferation, fluid secretion, and inflammation in the kidney [9]; P2X7 receptor is known to play an important role in renal IR injury by regulating T-cell expansion [10]. There are eight P2Y receptors (P2Y1, P2Y2, P2Y4, P2Y6, and P2Y11-14), widely distributed in the brain, heart, kidneys, and adipose tissues [8,11]. P2Y2 receptor (P2Y2R) is involved in glomerular cell proliferation and modulation of renal tubule function, including natriuresis [12,13,14]. P2Y2R activation is shown to decrease blood pressure and increase renal Na+ secretion, suggesting the potential use of P2Y2 agonism in the treatment of hypertension [15]. However, the role of P2Y2R during renal IR has not been fully characterized, and the proximal tubule-specific effect of P2Y2R has not yet been described. In this study, we investigated the role of P2Y2R on proximal tubule-specific effects using P2Y2R knockout (KO) mice and a selective P2Y2R agonist MRS2768 in in vivo and in vitro renal IR models.

## 2. Results

### 2.1. Renal IR Increased Plasma ATP Levels and Proximal Tubular P2Y2R Expression

**Figure 1:**
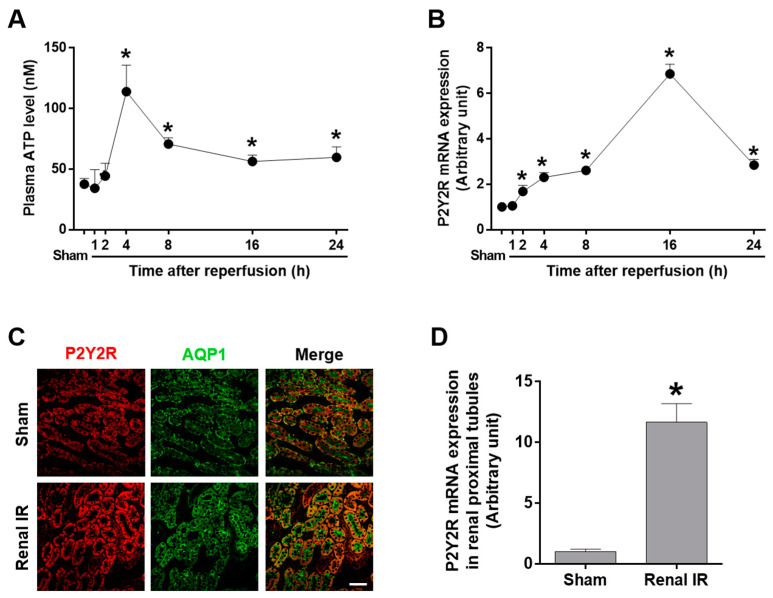
Extracellular ATP and proximal tubular P2Y2R expression are induced during renal IR injury. Blood and kidney tissue samples were collected from WT mice subjected to sham operation or 25 min of renal ischemia (n = 4–8). (**A**) Extracellular ATP released into the plasma was measured after the indicated times of reperfusion. (**B**) The mRNA expression of P2Y2R was determined by real-time RT-PCR analysis. (**C**) WT mice were subjected to a sham operation or 25 min of ischemia followed by 16 h of reperfusion. The proximal tubular expression of P2Y2R was confirmed by immunofluorescence staining using anti-P2Y2R and anti-AQP1 antibodies. (**D**) WT mice were subjected to a sham operation or 25 min of ischemia followed by 16 h of reperfusion. P2Y2R mRNA expression levels were determined by real-time PCR analysis in the proximal tubule fractions separated by Percoll density gradient centrifugation. Data are presented as mean  ±  SEM. * *p* < 0.05 vs. sham group. Scale bar, 100 μm.

Extracellular ATP is released from injured cells and acts as a danger signal, and the released ATP is recognized by purinergic receptors (P2X and P2Y) that involve various cellular defense responses, including inflammation, wound healing, and angiogenesis [16]. Firstly, extracellular ATP levels were measured in the plasma after 1, 2, 4, 8, 16, and 24 h of reperfusion (Figure 1A). The plasma ATP levels increased about 2-fold after 4 h of reperfusion compared to sham mice and then gradually decreased by 24 h. Renal mRNA expression of P2Y2R also increased as early as 2 h after IR, reached maximum levels 16 h after reperfusion, and decreased 24 h after reperfusion (Figure 1B). The proximal tubule is particularly vulnerable to IR injury due to its high rates of oxygen consumption and relative lack of endogenous antioxidant defenses [17]; the proximal tubule S3 segment is most susceptible to irreversible damage in AKI [18]. To investigate the localization of P2Y2R in proximal tubules, the kidney sections were double immunostained using anti-P2Y2R and anti-aquaporin 1 (AQP1, a proximal tubule cell marker) antibodies. P2Y2R was co-localized with AQP1, indicating its proximal tubular expression in sham-operated mice, and the localization of P2Y2R was not altered 16 h after renal IR (Figure 1C). The proximal tubular expression of P2Y2R was also confirmed by Percoll density gradient separation of kidney tissue lysates, where the mRNA levels of P2Y2R were elevated in the proximal tubule fraction 16 h after renal IR injury (Figure 1D) compared to sham. The P2Y2R expression was analyzed by the mRNA levels due to the lack of proper P2Y2R antibodies for Western blotting. The results suggest that IR-induced induction of ATP levels and P2Y2R expression may play an important role during reperfusion, particularly in the proximal tubules.

### 2.2. The Deficiency of P2Y2R Aggravated Renal IR Injury in Mice

**Figure 2:**
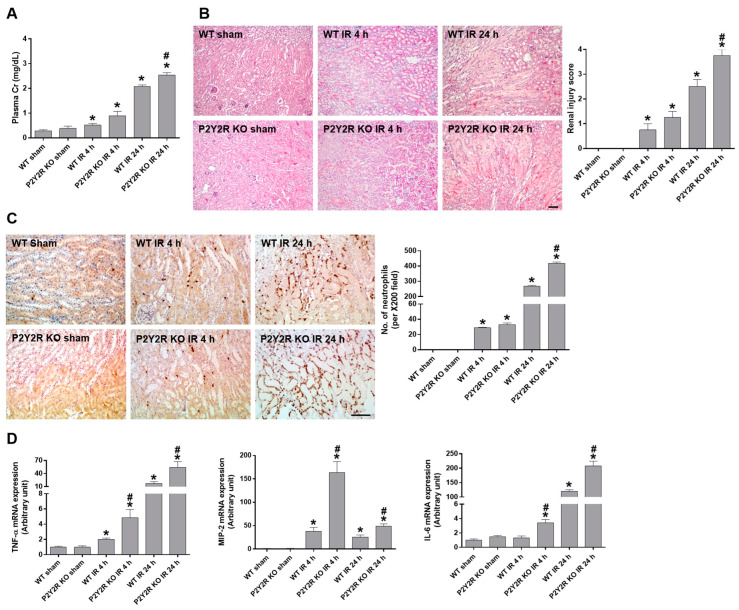
P2Y2R deficiency aggravates renal dysfunction and tubular damage during renal IR injury. Blood and kidney tissue samples were collected from WT and P2Y2R KO mice after a sham operation or 25 min of renal ischemia, followed by 4 or 24 h of reperfusion (n = 4–8). (**A**) Plasma creatinine levels and (**B**) representative H&E images of kidney sections are shown from the mice indicated; the extent of renal injury was scored. (**C**) Representative images of immunohistochemistry for neutrophil infiltration using an anti-Ly-6B.2 antibody are shown from the mice indicated; the number of stained neutrophils per ×200 field image was counted. (**D**) Relative mRNA expression of inflammatory cytokines (TNF-α, MIP-2, and IL-6) was determined by real-time PCR analysis in the kidney from the mice indicated. The data are presented as mean  ±  SEM. * *p* < 0.05 vs. WT sham group. ^#^ *p* < 0.05 vs. respective WT IR group. Scale bar, 100 μm.

To investigate the role of P2Y2R in the pathogenesis of renal IR, P2Y2R knockout (KO) mice were subjected to renal IR, and the structural and functional changes in the kidney were evaluated compared to wild-type (WT) mice. Plasma creatinine levels were significantly increased in mice after IR, and the levels were higher in KO mice compared to WT mice 24 h after IR (Figure 2A). Consistently, the histological staining of kidney tissue sections demonstrated that KO mice developed severe injury with much higher renal injury scores compared to WT mice (Figure 2B). The results indicate that the P2Y2R deficiency aggravates the IR-induced renal injury, implying that P2Y2R may play a protective role against the pathogenesis of renal IR.

### 2.3. The Deficiency of P2Y2R Increased Renal Inflammation After Renal IR Injury

P2Y2R is considered an essential chemotactic receptor for neutrophils [19] and is activated by the released ATP from damaged cells after renal IR (Figure 1A). Thus, we investigated whether the activation of P2Y2R by ATP recruits neutrophil infiltration after IR. The number of neutrophils recruited to renal tubular cells was evaluated by immunohistochemical staining of kidney sections using an anti-Ly-6B.2 antibody. As consistent with previous data, 24 h of reperfusion showed more neutrophil recruitment than 4 h, and KO mice had significantly higher neutrophil infiltration, compared to WT mice 24 h after IR (Figure 2C). The mRNA expression of tumor necrosis factor-α (TNF-α), macrophage inflammatory protein-2 (MIP-2), and interleukin-6 (IL-6) was similar in the sham of WT and KO mice; however, their levels were significantly increased and higher in KO mice compared to WT after renal IR (Figure 2D). The results suggest that P2Y2R protects against renal IR-induced inflammatory responses.

### 2.4. The Deficiency of P2Y2R Increased Renal Apoptosis After Renal IR Injury

**Figure 3:**
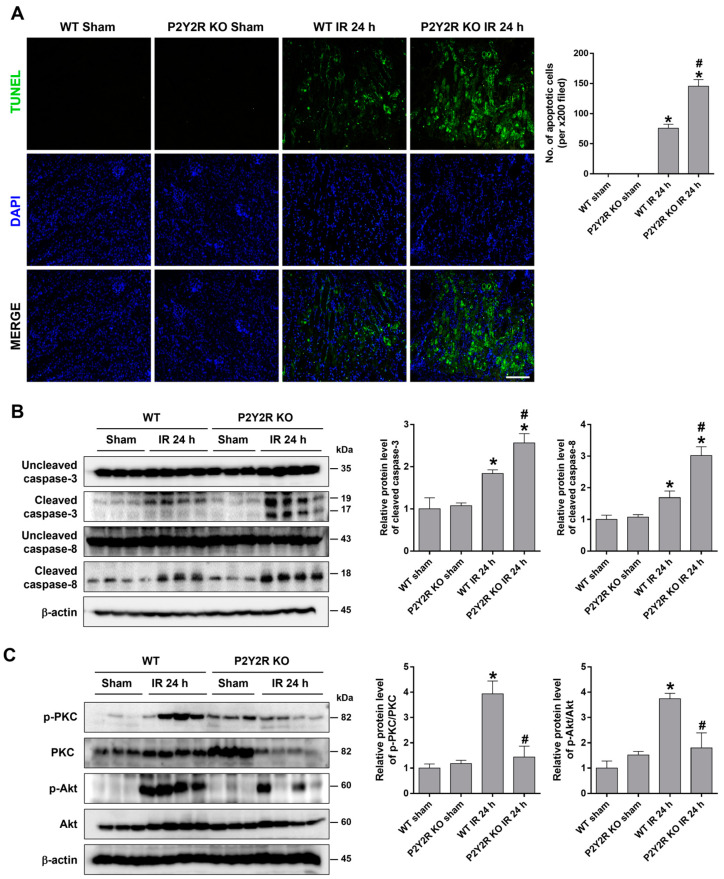
P2Y2R deficiency aggravates renal tubular apoptosis during renal IR injury. Kidney tissues were from WT and P2Y2R KO mice after sham operation or 25 min of renal ischemia, followed by 24 h of reperfusion (n = 4–8). (**A**) Representative TUNEL images in the outer medulla and the quantification of apoptotic cells are shown. (**B**) Representative immunoblots for uncleaved and cleaved forms of caspase-3, -8, and a loading control β-actin are shown; the quantitative analysis is from five independent experiments. (**C**) Representative immunoblots for p-PKC, PKC, p-Akt, Akt, and β-actin are shown. Data are presented as mean ± SEM. * *p* < 0.05 vs. WT sham group. ^#^ *p* < 0.05 vs. WT IR group. Scale bar, 100 μm.

To investigate whether P2Y2R deficiency aggravates renal apoptosis after renal IR, TUNEL assays were performed in the kidney section of WT and KO mice. After 24 h of reperfusion, the mice exhibited TUNEL-positive apoptotic cells prominently in the corticomedullary junction; the degree of apoptosis was more severe in KO mice than in WT mice (Figure 3A). The apoptosis was also evaluated by Western blot analysis. The cleaved levels of caspase-3 and caspase-8 were increased after 24 h of reperfusion; the levels were significantly higher in KO mice than in WT mice (Figure 3B). The downstream signaling kinases of P2Y2R have been extensively studied, and PKC and Akt are critical regulators of renal cell survival. The phosphorylated PKC and Akt levels were significantly increased after 24 h of reperfusion; the levels were reduced in KO mice compared to WT (Figure 3C). The results suggest that P2Y2R activation and the downstream PKC and Akt signaling may reduce IR-induced apoptosis.

### 2.5. P2Y2R Activation Reduced Plasma Creatinine, Tubular Injury, Renal Inflammation, and Apoptosis After Renal IR Injury

**Figure 4:**
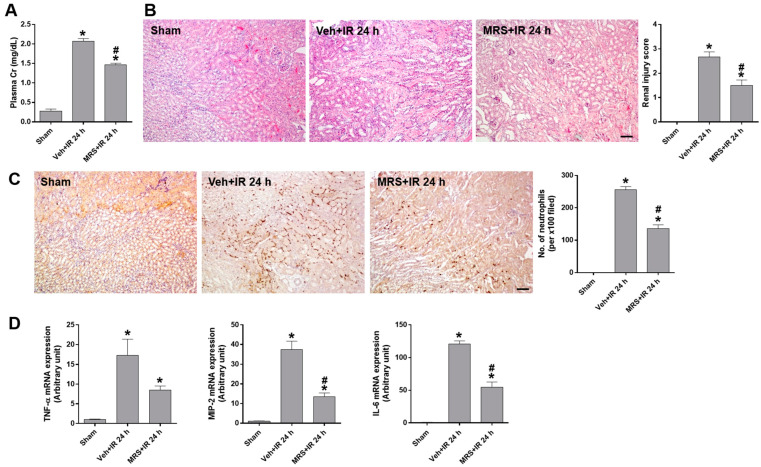
MRS2768, a P2Y2R agonist, improves renal function and ameliorates tubular damage during renal IR injury (n = 4–8). WT mice were treated with MRS2768 (MRS), 1 h before renal IR. (**A**) Plasma creatinine and (**B**) representative H&E images of kidney sections are shown from the mice indicated; the extent of renal injury was scored. (**C**) Representative images of immunohistochemistry for neutrophil infiltration using anti-Ly-6B.2 antibody are shown from the mice indicated; the number of stained neutrophils per ×100 field image was counted. (**D**) Relative mRNA expression of TNF-α, MIP-2, and IL-6 was determined by real-time PCR analysis. Data are presented as mean ± SEM. * *p* < 0.05 vs. sham group. ^#^ *p* < 0.05 vs. IR group. Scale bar, 100 μm.

**Figure 5:**
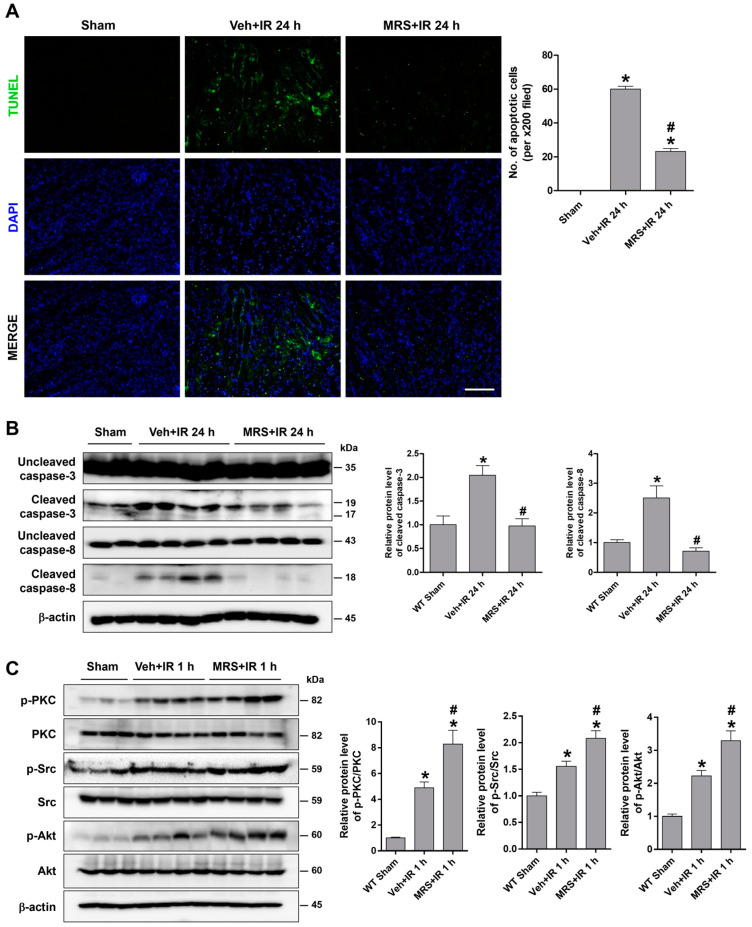
MRS2768, a P2Y2R agonist, attenuates renal tubular apoptosis and activates PKC, Src, and Akt signaling kinases during renal IR injury. WT mice were treated with MRS2768 (MRS), 1 h before renal IR (n = 4–8). (**A**) Representative TUNEL images in the outer medulla and the quantification of apoptotic cells are shown. (**B**) Representative immunoblots for pro- and cleaved forms of caspase-3, -8 and a loading control β-actin and the quantitative analysis are shown. (**C**) Kidney tissues were collected after 1 h reperfusion of ischemic mice. Representative immunoblots for p-PKC, PKC, p-SRC, SRC, p-Akt, Akt, and β-actin are shown. Data are presented as mean ± SEM. * *p* < 0.05 vs. sham group. ^#^ *p* < 0.05 vs. IR group. Scale bar, 100 μm.

To determine the effect of P2Y2R activation on renal IR injury, a selective agonist MRS2768 was injected intraperitoneally at 25 μg/kg body weight 1 h before ischemia. Plasma creatinine levels were increased 24 h after IR, but this increase was inhibited by MRS2768 treatment (Figure 4A). The IR-induced tubular injury and necrosis were also reduced by MRS2768 treatment (Figure 4B). Consistently, neutrophil infiltration in ischemic tissues after renal IR was significantly increased but reduced by MRS2768 treatment (Figure 4C); MRS2768 attenuated the increased mRNA levels of proinflammatory cytokines (TNF-α, MIP-2, and IL-6) after IR (Figure 4D). In addition, the tubular apoptosis was determined by TUNEL staining, and the number of apoptotic cells was increased after IR but reduced by MRS2768 treatment (Figure 5A). Consistently, the levels of cleaved caspase-3 and -8 were significantly reduced by MRS2768 treatment in mice subjected to IR (Figure 5B). We then evaluated P2Y2R activation and the downstream signaling kinase activities of the PKC, Src, and Akt pathways, which have been shown to be involved in cell survival [20]. The phosphorylation of PKC, Src, and Akt was significantly induced 1 h after renal IR, compared to sham; their levels were further increased by MRS2768 treatment (Figure 5C). The results indicate that P2Y2R activation through PKC, Src, and Akt may attenuate the IR-induced tubular inflammation and apoptosis and prevent renal dysfunction.

### 2.6. P2Y2R Activation Induced by MRS2768 or ATP Is Associated with Phosphorylation of PKC, Src, and Akt in Human Proximal Tubular Cells

**Figure 6:**
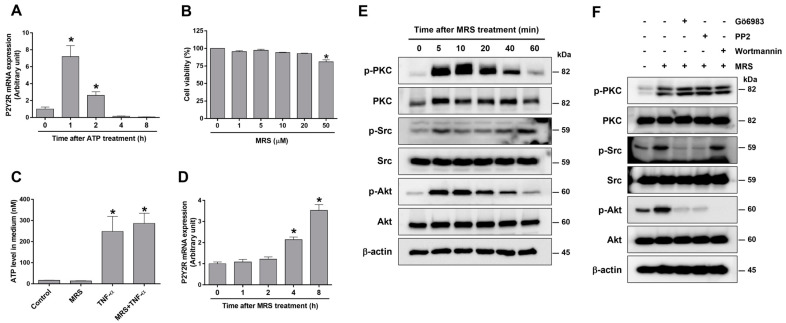
MRS2768 increases the P2Y2R expression and activates PKC, Src, and Akt in human proximal tubular HK-2 cells. (**A**) HK-2 cells were treated with ATP (10 μM) for the indicated times, and P2Y2R mRNA levels were assessed by real-time PCR analysis. (**B**) Cells were treated with MRS2768 (MRS, 1, 5, 10, 20, 50 μM) for 24 h, and cell viability was determined by MTT assay. (**C**) Cells were pretreated for 1 h with MRS2768 (20 μM) or vehicle and treated with TNF-α (20 ng/mL) for 5 min, then the released ATP levels in the media were measured. (**D**) Cells were pretreated with MRS2768 (20 μM) for the indicated times, and P2Y2R mRNA levels were assessed by real-time PCR analysis. (**E**) Cells were treated with MRS2768 for the indicated times, and cell lysates were subjected to Western blot analysis using p-PKC, PKC, p-SRC, SRC, p-Akt, Akt, and β-actin antibodies. (**F**) Cells were pretreated with Gö6983 (10 μM), PP2 (10 μM), and wortmannin (100 nM) for 1 h and treated with MRS2768 for 5 min. The cell lysates were subjected to Western blot analysis with antibodies against p-PKC, PKC, p-Src, Src, p-Akt, Akt, and β-actin. Each experiment was repeated more than 3 times. Data are presented as mean ± SEM. * *p* < 0.05 vs. control group.

To determine the effect of P2Y2R activation specific to proximal tubular cells, HK-2 cells were treated with MRS2768. First, we examined whether ATP induces P2Y2R expression. The cells were treated for different times (1, 2, 4, or 8 h) with 10 μM of ATP and measured the P2Y2R mRNA levels; the levels were increased 1 and 2 h after ATP treatment (Figure 6A). The cytotoxic concentration of MRS2768 was determined by MTT assay in cells treated with MRS2768 (1, 5, 10, 20, or 50 μM) for 24 h; cell viability was decreased at 50 μM of MRS2768 (Figure 6B). Next, we investigated whether the extracellular ATP is released into the medium by MRS2768 or TNF-α treatment (Figure 6C). ATP levels were not changed by MRS2768 alone but were significantly increased by TNF-α and combined treatment of TNF-α and MRS2768. To examine whether MRS2768 increases P2Y2R expression, the mRNA levels were measured after different times (1, 2, 4, or 8 h) of MRS2768 treatment. A significant induction was found 4 and 8 h after treatment (Figure 6D). In addition, the phosphorylation of PKC, Src, and Akt was significantly increased after MRS2768 treatment in HK-2 cells (Figure 6E). The induction of p-PKC and p-Akt was found as early as 5 min, lasted, and tapered at 60 min; p-Src showed two peaks of induction at 5 and 60 min after treatment. The results indicate that the P2Y2R activation induced by MRS2768 or ATP released upon inflammation may be associated with the activation of PKC, Src, and Akt signaling.

Then, we confirmed the specific activation of PKC, Src, and Akt signaling kinases by MRS2768 treatment using the respective inhibitors, Gö6983, PP2, and wortmannin. The inhibitors were pretreated 1 h prior to MRS2768, and the phosphorylated levels of PKC, Src, and Akt were measured by Western blot analyses (Figure 6F). MRS2768 increased p-PKC, p-Src, and p-Akt, suggesting that P2Y2R activation increased these signaling kinases. Interestingly, p-Akt levels were attenuated by Gö6983, PP2, and wortmannin, whereas p-Src levels were attenuated by Gö6983 and PP2, but unaffected by wortmannin. The results suggest that Src and Akt are the downstream kinases of PKC upon MRS2768 treatment.

### 2.7. P2Y2R Activation Decreased TNF-α-Stimulated Cell Death via PKC, Src, and Akt Pathways in Human Proximal Tubular Cells

**Figure 7:**
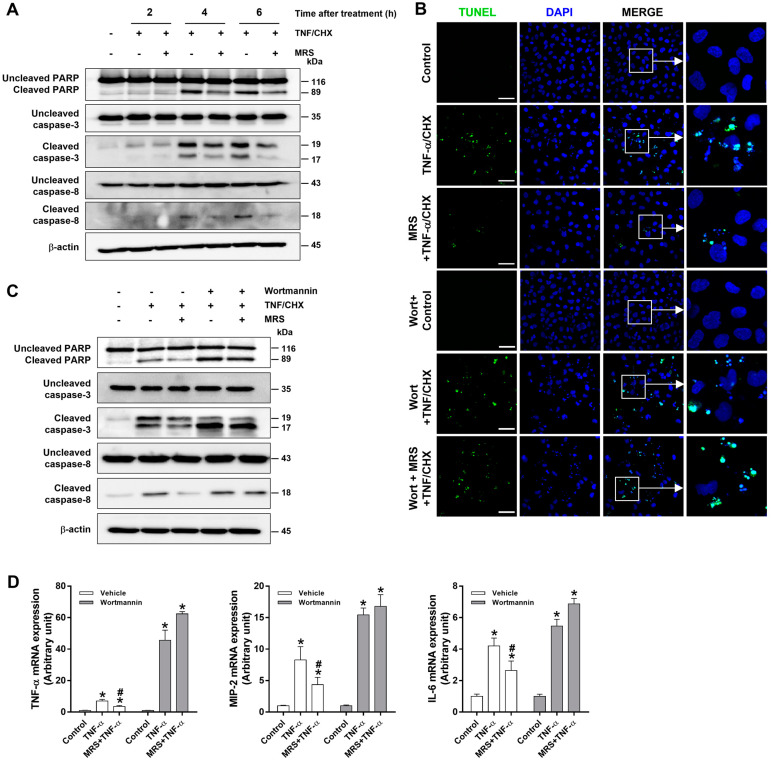
Activation of P2Y2R attenuates apoptosis induced by TNF-α and cycloheximide in HK-2 cells. (**A**) HK-2 cells were pretreated for 1 h with MRS2768 (MRS, 20 μM) and treated with TNF-α (20 ng/mL) and cycloheximide (CHX, 10 μg/mL) for 2, 4, or 6 h. The cell lysates were subjected to Western blot analysis using antibodies against PARP, caspase-3, -8, and β-actin. (**B**) Cells were pretreated with wortmannin (100 nM) and/or MRS2768 (20 μM) for 1 h and then were treated with TNF-α (20 ng/mL) and CHX (10 μg/mL) for 6 h. The apoptotic cells were stained by TUNEL assay. (**C**) The cell lysates were subjected to Western blot analysis with antibodies against PARP, caspase-3, -8, and β-actin. (**D**) The mRNA expression of TNF-α, MIP-2, and IL-6 was analyzed by real-time PCR. Each experiment was repeated more than 3 times. Data are presented as mean ± SEM. * *p* < 0.05 vs. control group, ^#^ *p* < 0.05 vs. TNF-α/CHX-treated or TNF-α-treated group. Scale bar, 100 μm.

We investigated whether P2Y2R activation reduces apoptotic cell death in HK-2 cells. First, the cells were exposed to TNF-α plus cycloheximide for the induction of apoptosis [21,22]. Then, we demonstrated whether P2Y2R activation reduces the levels of cleaved caspases and poly (ADP-ribose) polymerase (PARP), which are the markers of apoptosis [23]. Incubation of HK-2 cells with TNF-α plus cycloheximide for 4 and 6 h resulted in a dramatic increase in cleaved PARP, caspase-3, and caspase-8, and the cleaved levels were attenuated by MRS2768 pretreatment (Figure 7A). The cells exposed to TNF-α plus cycloheximide for 16 h showed typical apoptotic features of nuclear fragmentation and chromatin condensation in TUNEL assays; MRS2768 pretreatment decreased these abnormally shaped nuclei and reduced the number of TUNEL-positive cells (Figure 7B). To determine whether the PI3K/Akt pathway is involved, cells were treated with wortmannin, a PI3K/Akt inhibitor, prior to the induction of apoptosis. Treatment of wortmannin abolished the protective effect of MRS2768 and showed apoptotic features (Figure 7B). In addition, wortmannin abolished the anti-apoptotic effect of MRS2768 by exacerbating the levels of cleaved PARP, caspase-3, and caspase-8 in cells incubated with TNF-α plus cycloheximide (Figure 7C). Consistently, MRS2768 pretreatment reduced the mRNA expression levels of proinflammatory cytokines (TNF-α, MIP-2, and IL-6) in cells incubated with TNF-α (Figure 7D, white bars). Wortmannin abolished the protective effect of MRS2768 and further increased the cytokine levels (Figure 7D, gray bars). The results indicate that P2Y2R activation protects proximal tubular cells from apoptotic stress and TNF-α stimulation through the PI3K/Akt signaling pathway.

## 3. Discussion

The present study demonstrates that P2Y2R activation protects against renal IR injury. The major findings are as follows: First, renal IR injury resulted in a significant upregulation of P2Y2R expression in proximal tubular cells with increased extracellular ATP levels. Second, P2Y2R KO mice showed an aggravated pathology of IR compared to WT mice. Third, pretreatment of MRS2768, a selective P2Y2R agonist, protected the kidney against renal IR injury in vivo and attenuated apoptosis and inflammation of renal proximal tubular cells in vitro. Fourth, P2Y2R activation induced by MRS2768 is through the PKC, Src, and Akt signaling pathway. Therefore, the proximal tubular activation of P2Y2R may contribute to its protective effect against renal IR injury.

Irreversible tubular apoptosis induced by IR contributes to renal damage and dysfunction and impairs tissue repair processes [24]; however, the molecular mechanisms of tubular apoptosis and possible therapeutic targets are not fully understood. In the present study, we hypothesized that ATP released from damaged cells plays an important role in the pathogenesis of renal IR, particularly activating P2Y2R in proximal tubular cells. Extracellular ATP acts as a danger signal to stimulate purinergic receptors of P1, P2X, and P2Y, which are differentially expressed in the body [25,26]. P2Y2R has been recognized as an essential chemotactic receptor for neutrophils [27]; P2Y2R recruits neutrophils to the lungs in a mouse model of sepsis [28] and mediates chemotaxis of dendritic cells and eosinophils in allergic lung inflammation [29]. P2Y2R also promotes neutrophil infiltration and hepatocyte death in mice with acute liver injury [30]. In contrast, ATP released from dying cells during acute kidney injury induces the proliferation of neighboring tubular cells, promoting wound closure via the activation of Akt [31]. Moreover, P2Y2R activation protects cardiomyocytes from hypoxia and reduces post-ischemic myocardial damage [32]. In this study, we demonstrate that extracellular ATP and subsequent activation of P2Y2R protect the kidney against renal IR injury by attenuating tubular apoptosis and inflammation via the PI3K/Akt pathway.

P2Y2R is expressed throughout the kidney, including glomerulus, podocytes, proximal and distal tubules, and collecting ducts [25,26]. However, most studies have focused on the impaired regulation of renal microvascular function by P2Y2R in association with diseases such as hypertension, diabetes, and sepsis [33]. Here, we investigated the role of P2Y2R specific to proximal tubules in renal IR injury. We found that the stimulation of P2Y2R inhibits apoptosis through PKC/Src/Akt pathway. Tempol-enhanced Akt activity has previously been shown to protect the kidney from IR injury through Nrf2-mediated transcriptional activation of antioxidant genes [34]. Previous studies have shown that P2Y2R activation triggers the Gαq-dependent activation of phospholipase Cβ (PLCβ) to generate inositol 1,4,5-trisphosphate (IP3) and diacylglycerol (DAG) and upregulates intracellular [Ca^2+^] and PKC activity, respectively. Activation of PKC protects against cerebral and myocardial IR injury through cell survival Akt signaling [35,36]. PKC and Src activation induced by G-protein-coupled receptors are known to activate tyrosine kinases, such as the epidermal growth factor (EGF) receptor, leading to activation of downstream signaling pathways, MAPK/ERK (mitogen-activated protein kinase–extracellular signal-regulated kinase), PI3K/Akt, and JAK/STAT (Janus kinase/signal transducers and activators of transcription) pathways. The consequence of these signaling events is the translocation of external/cytosolic signals to the nucleus, triggering the transcription of genes for cell proliferation, differentiation, and migration [37]. Thus, Src inhibition has been shown to protect against renal damage by regulating multiple cellular signaling molecules associated with tubular cell death. Src also interacts directly with the C-terminal proline-rich motif of Akt for its phosphorylation and subsequent activation [38]. The serine/threonine kinase Akt is important for the cell survival pathway in many cell types [39,40,41]. In particular, Akt inhibits cell death by preventing cytochrome c release from mitochondria [42]. Akt also inhibits activation of caspase-9 and -3 by posttranslational modification of downstream factors of cytochrome c and regulates proapoptotic factors [5].

Inflammation is a major pathogenic factor in ischemic acute kidney injury through leukocyte infiltration into the renal parenchyma to exacerbate tubular cell death [43,44,45,46]. During reperfusion, infiltration of neutrophils, macrophages, and lymphocytes promotes the release of proinflammatory cytokines and chemokines and exacerbates free radical-mediated tubular injury [47]. The damaged proximal tubular epithelial cells also upregulate adhesion molecules to facilitate leukocyte–endothelial adhesion; early expression of renal TNF-α contributes to neutrophil infiltration during reperfusion by increasing the expression of adhesion molecules [48,49]. P2Y2R deficiency exacerbates renal inflammation and tubular damage in the mouse model of chronic kidney disease [50]. In addition, P2Y2R regulates IL-6 and IL-8 release from renal epithelial cells in bacteria-induced urinary tract infections, suggesting its contribution to innate immune responses [51,52]. In this study, P2Y2R activation reduced inflammatory responses in both in vivo and in vitro experimental models, as evidenced by decreased neutrophil infiltration and expression of proinflammatory cytokines.

A recent study showed sex-specific roles of P2Y2R in glucose homeostasis. The study on glucose tolerance during fasting and acute inflammation demonstrates that P2Y2R has a significant role in males, while its impact in females is minimal [53]. The sex-specific difference was also reported during the recovery after IR. Females show an improved recovery from kidney transplantation compared with males [54]. Investigating both sexes could enhance our understanding of differential hormonal and immune responses, and effective pharmacological treatment should benefit patients of both sexes.

In this study, we measured extracellular ATP levels in WT mice to investigate the amount of ATP released from damaged kidneys following IR. The extracellular ATP as a signaling molecule activates P2Y2R and downstream pathways. However, P2Y2R KO or activation by agonist (MRS2768) is able to affect extracellular ATP levels following IR and subsequent cellular injury processes. Further analysis of the extracellular ATP would improve our understating of the role of P2Y2R and associated signaling following IR.

In conclusion, P2Y2R activation induced by extracellular ATP protects the kidney from IR-induced inflammation and proximal tubular apoptosis. We suggest P2Y2R as a cytoprotective receptor through signaling associated with PKC, Src, and Akt pathways. These support the potential role of selective P2Y2R agonists to protect against various types of perioperative acute kidney injury.

## 4. Materials and Methods

### 4.1. The Animals

WT C57BL/6 mice were purchased from Koatech (Pyeongtaek, Republic of Korea), and P2Y2R KO mice on C57BL/6 background (B6.129P2-*P2ry2^tm1Bhk^*/J) were obtained from Jackson Laboratory (Bar Harbor, ME, USA). All mice were maintained in the animal facility of Gyeongsang National University. All animal experiments were approved by the Institutional Board of Animal Research at Gyeongsang National University (GNU-180615-M0028) and conducted in accordance with the National Institutes of Health guidelines for laboratory animal care. Mice were maintained with a 12 h light/dark cycle and provided freely with water and standard chow.

### 4.2. Mouse Model of Renal IR Injury

Male mice (7 weeks old) were habituated for 1 week and randomly divided as follows: (1) WT sham-operated mice (n = 4); (2) WT mice subjected to renal IR (n = 8); (3) KO sham-operated mice (n = 4); and (4) KO mice subjected to renal IR (n = 8). Mice were also divided as follows: (1) sham-operated mice (n = 4); (2) mice subjected to renal IR (n = 8); and (3) mice pretreated 1 h prior to renal IR intraperitoneally with MRS2768 (n = 8; Tocris Bioscience, Bristol, UK) at the dose of 25 μg/kg. The mice were anesthetized with zoletil (0.5 mg/kg; Virbac Laboratories, Carros, France) and placed supine on a heating pad under a heat lamp to maintain body temperature. After the abdominal incision, a microvascular clamp was placed on the left renal pedicle for 25 min, and the right kidney was ligated and removed. The mice remained hydrated with warm saline during the ischemic period, and the incision was sutured after removing the clamp. The sham-operated mice were subjected to right nephrectomy without clamping. Mice were sacrificed at the indicated times after reperfusion by CO_2_ euthanasia, and blood and kidney tissues were collected. The kidney tissues were rapidly frozen in liquid nitrogen for storage at −80 °C or fixed in 10% buffered formalin. Blood was from an inferior vena cava by using a heparinized syringe, centrifuged at 3000× *g* for 20 min. Plasma creatinine levels were measured by using Pure Auto S CRE-N (Daiichi Sankyo, Tokyo, Japan).

### 4.3. Cell Culture

HK-2 human proximal tubular epithelial cells were purchased from ATCC (CRL-2190) and grown in DMEM/F12 medium (1:1 mixture of Dulbecco’s modified Eagle medium and Ham’s F-12 medium), supplemented with 10% (*v*/*v*) fetal bovine serum (Hyclone Laboratories, Logan, UT, USA) and 1% penicillin/streptomycin in a 5% CO_2_ incubator at 37 °C. Cells were treated with MRS2768, Gö69838 (Sigma-Aldrich, St. Louis, MO, USA), PP2 (Sigma-Aldrich), and wortmannin (Sigma-Aldrich) as indicated in figure legends. Recombinant human TNF-α (R&D Systems, Minneapolis, MN, USA) and cycloheximide (Sigma-Aldrich) were used to induce the proximal tubular cell injury as indicated in figure legends.

### 4.4. Cell Viability

Cell viability was assessed by 3-(4,5-dimethylthiazol-2-yl)-2,5-diphenyltetrazolium bromide (MTT) assay. The cells were incubated with MTT solution (final 0.1 mg/mL) and incubated at 37 °C for 4 h. Then, the supernatant was removed, and formazan crystals were dissolved in dimethyl sulfoxide. Absorbance at 570 nm was measured using an Infinite 200 microplate reader (Tecan Austria GmbH, Grödig, Austria).

### 4.5. Isolation of Mouse Kidney Proximal Tubules

Mouse kidney proximal tubules were isolated by using a method as described [55,56]. After 15 h of reperfusion or sham operations, the kidneys were immediately harvested and washed in sterile, ice-cold, and 95% O_2_/5% CO_2_ equilibrated Krebs–Henseleit saline (KHS, pH 7.4), which contains 119 mM of NaCl, 4.7 mM of KCl, 1.9 mM of CaCl_2_, 1.2 mM of KH_2_PO_4_, 1.2 mM of MgSO_4_∙7H_2_O, and 25 mM of NaHCO_3_. Each kidney was decapsulated and bisected, and the inner medullary portion was excised. The cortical and outer medullary regions were pulverized to mix in a solution of 30 mL of KHS containing 1 mg/mL of collagenase type I (Thermo Fisher Scientific, Waltham, MA, USA). The solution was bubbled with 95% O_2_/5% CO_2_ during incubation for 30 min at 37 °C. The digested solution was filtered through a 210 μm mesh sieve (Thermo Fisher Scientific) and subjected to Percoll (Sigma-Aldrich) density gradients. The filtered solution was slowly added to the top layer of a centrifuge tube containing 30 mL of 45% Percoll and 5 mL of 90% Percoll solution. Then, the tubes were centrifuged at 20,000× *g* and 4 °C for 30 min, and centrifugation resulted in 4 separated bands; the proximal tubules in the 3rd layer were extracted by aspiration and centrifuged at 1500× *g* for 2 min at room temperature to remove the Percoll, for further analysis.

### 4.6. Extracellular ATP Level Measurement

Extracellular ATP levels were measured in fresh mouse plasma or supernatant collected from cell culture media by a bioluminescent detection method using the ENLITEN ATP assay kit (Promega, Madison, WI, USA) according to the manufacturer’s instructions. The ATP levels were determined by Glomax^®^ 20/20 luminometer (Promega) and calculated based on an ATP standard curve.

### 4.7. H&E Staining and TUNEL Assay

Kidney tissues were fixed in 10% formalin for 24 h, processed for paraffin embedding, and sectioned at 5 µm. Then, the sections were stained with hematoxylin and eosin (Sigma-Aldrich) by a standard protocol. The renal injury scores were determined semi-quantitatively, as previously described [57]. TUNEL assay was performed by using an in situ cell death detection kit (Roche Molecular Biochemicals, Mannheim, Germany) according to the manufacturer’s instructions. The number of TUNEL-positive cells was counted from five microscopic fields (200×) per section from each group (n = 3). H&E images were captured by a CKX41 light microscope (Olympus, Tokyo, Japan), and TUNEL images were captured by a Fluoview FV1000 confocal microscope (Olympus). All images were quantified by ImageJ version 1.54g (National Institutes of Health, Bethesda, MD, USA).

### 4.8. Immunohistochemistry

The sections were deparaffinized, rehydrated, and antigen-retrieved in sodium citrate buffer (10 mM, pH 6.0) for 20 min. Endogenous peroxidase activity was blocked with 0.3% hydrogen peroxide, and nonspecific binding was blocked with 10% normal goat serum. The sections were incubated with a primary anti-Ly-6B.2 antibody (Bio-Rad, Hercules, CA, USA) overnight at 4 °C and with a biotinylated secondary antibody (Vector Laboratories, Burlingame, CA, USA) for 1 h at room temperature. The sections were incubated in avidin–biotin–peroxidase complex solution (ABC solution; Vector Laboratories) for 30 min and developed using a 3,3′-diaminobenzidine (DAB) Peroxidase Substrate Kit (Vector Laboratories). Then, the sections were counterstained with hematoxylin, and the images were obtained by a CKX41 light microscope (Olympus).

### 4.9. Immunofluorescence Staining

The kidney sections were blocked in 2.5% normal horse serum and incubated with anti-P2Y2R and AQP1 antibodies (Santa Cruz Biotechnology, Dallas, TX, USA) overnight at 4 °C. After washing, the sections were incubated with the corresponding Alexa 594- and Alexa 488-conjugated secondary antibodies (Vector Laboratories) for 1 h at room temperature. The fluorescent images were obtained using a Fluoview 1000 (IX-81) confocal microscope (Olympus).

### 4.10. Western Blot Analysis

Kidney tissues and HK-2 cells were homogenized in ice-cold RIPA buffer with protease inhibitors (Thermo Fisher Scientific), sonicated, and incubated for 20 min on ice. The lysates were separated and transferred to polyvinylidene difluoride membranes. After blocking in 5% skim milk or 3% bovine serum albumin for 1 h at room temperature, the membranes were incubated overnight at 4 °C in primary antibodies as follows: p-PKC, p-Akt, Akt, Src, caspase-3, caspase-8, PARP (Cell Signaling Technology, Danvers, MA, USA); PKC and P2Y2R (Santa Cruz Biotechnology); p-Src (Invitrogen, Carlsbad, CA, USA); and β-actin (Sigma-Aldrich). After washing, the membranes were incubated with the appropriate horseradish peroxidase-conjugated secondary antibodies (Bio-Rad) for 1 h at room temperature. Then, the membranes were developed using a Clarity™ Western ECL Substrate (Bio-Rad), and the protein band intensity was analyzed by the ChemiDoc XRS+ System (Bio-Rad).

### 4.11. Quantitative Reverse Transcription PCR

Total RNA was extracted with Trizol (Invitrogen) and converted to cDNA using the RevertAid Reverse Transcription System (Thermo Fisher Scientific) according to the manufacturer’s instructions. Real-time PCR analysis was performed with a CFX Connect real-time PCR System using iQ SYBR Green Supermix (Bio-Rad). PCR was performed with an initial preincubation step for 10 min at 95 °C, followed by 40 cycles of 95 °C for 10 s, annealing at 60 °C for 10 s, and extension at 72 °C for 10 s. The relative mRNA levels were normalized to those of glyceraldehyde 3-phosphate dehydrogenase (GAPDH) by 2(-delta delta C(T)) method [58]. The list of primer sequences is shown in Table 1.

### 4.12. Statistical Analysis

Statistical difference was assessed by a two-tailed student *t*-test to compare two groups or by one-way analysis of variance (ANOVA) followed by Tukey’s post hoc multiple comparison tests for multiple groups. All values are expressed as means ± standard error of the mean (SEM). A *p* value < 0.05 was considered statistically significant.

## Figures and Tables

**Table 1:** The primer sequences used for real-time PCR analysis in this study.

Gene		Forward Primers (5′-3′)	Reverse Primers (3′-5′)
Mouse	*P2Y2R*	ACCACCTACATGTTTCACC	GGCGTAGTAATAAACCAACA
	*Tnfα*	CATATACCTGGGAGGAGTCT	GAGCAATGACTCCAAAGTAG
	*Mip-2*	AGAGGGTGAGTTGGGAACTA	GCCATCCGACTGCATCTATT
	*IL-6*	GACTTCCATCCAGTTGCCTTCTTG	GGTATCCTCTGTGAAGTCTCCTCT
	*Gapdh*	GTGGCAAAGTGGAGATTGTTG	TTGACTGTGCCGTTGAATTTG
Human	*P2y2r*	GTGCTCTACTTCCTGGCT	CTGAAGTGTTCTGCTCCTAC
	*Tnfα*	TGCTGCAGGACTTGAGAAGA	GGCTACATGG-GAACAGCCTA
	*Mip-2*	GCATCGCCCATGGTTAAGA	TCAGGAACAGCCACCAATAAG
	*IL-6*	CCAGGAGAAGATTCCAAAGATGTA	CGTCGAGGATGTACCGAATTT
	*Gapdh*	GGTGTGAACCATGAGAAGTATGA	GAGTCCTTCCACGATACCAAAG

## Data Availability

The data that support the findings of this study are available from the corresponding author upon reasonable request.
